# Endobronchial Carcinoid Tumor in a Girl with Initial Histologic Diagnosis of Leiomyoma

**Published:** 2015-09-01

**Authors:** Muhammad Arshad, Mishraz Shaikh, Mehmood-ul Haq, Syed Waqas Ali

**Affiliations:** 1 Department of Paediatric Surgery, Liaquat National Hospital, Karachi, Pakistan; 2 Department of Thoracic Surgery, Liaquat National Hospital, Karachi, Pakistan; 3 Department of Paediatric Surgery, National Institute of Child Health, Karachi, Pakistan

**Keywords:** Endobronchial carcinoid, Leiomyoma, Pediatric

## Abstract

Endobronchial tumors represent the rarest cause of airway obstruction in pediatric population. Due to rarity of the condition, a high index of suspicion is required for early diagnosis. We report a patient in whom diagnostic bronchoscopic biopsy was reported as leiomyoma while post resection histopathology showed an atypical carcinoid.

## INTRODUCTION

Bronchial carcinoids are rare tumors and characterized as low grade malignant tumors with metastatic and invasion potential. They arise from neuroendocrine Kulschitzky cells found in basal layer of bronchial epithelium. Based on presence/absence of necrosis and normal/raised mitotic index; carcinoid tumors are classified in to typical (10%) or atypical (90%) subgroups. An elevated mitotic index (2 mitoses/ 10 HPF) associated with necrosis is consistent with an atypical carcinoid tumor and with a worse prognosis as compared to typical tumors. [1] Herein one such case is reported.

## CASE REPORT

A 13-year-old girl referred from other city with the complaints of choking and hemoptysis for last five months. In her native city she was initially managed as a case of pulmonary tuberculosis and took anti-tuberculous therapy but symptoms did not subside. She underwent bronchoscopy and found to have an obstructing mass in left main stem bronchus. Due to some technical issues related to biopsy sampling, she was referred to our institute.

On examination, there was no sign of respiratory distress. Percussion note was dull on left side with no air entry on auscultation. Chest x-ray showed collapsed left lung. CT scan chest showed a 2.5 cm x 2 cm mass in left upper lobe bronchus causing complete collapse of left lung (Fig. 1). Bronchoscopy showed an obstructing lesion in left main bronchus (Fig. 2). A biopsy was taken which revealed spindle shaped lesion without any atypia favoring leiomyoma. The girl underwent a left thoracotomy and pneumonectomy in collaboration with a cardiothoracic surgeon. The tumor was completely excised. Upper lobe was found completely involved by tumor while lower lobe cannot be salvaged due to extension into main stem bronchus (Fig. 3). Histopathology showed infiltrating carcinoid tumor staining positive for chromogranin. Ki-67 showed increased proliferative index. Postoperatively, the patient had an uneventful course. Her chest drain was removed on first postoperative day. She was discharged on 5th postoperative day and is doing fine on follow-up.

**Figure:**
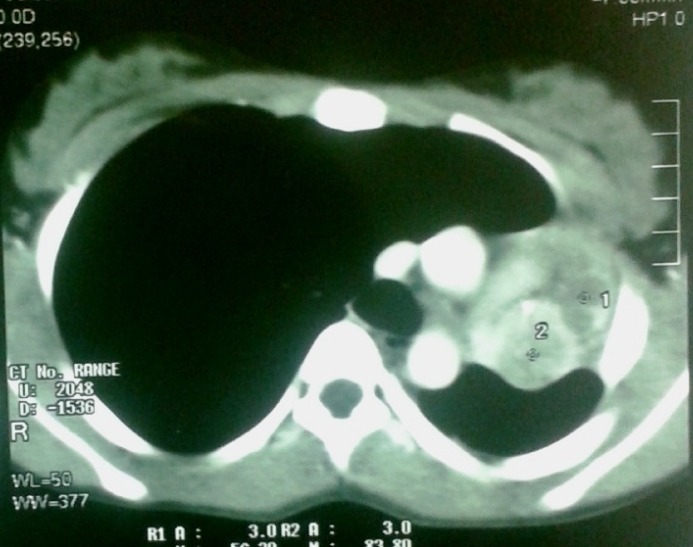
Figure 1:CT scan of chest showing obstruction of left main bronchus with resultant collapse of left lung and mediastinal shift.

**Figure:**
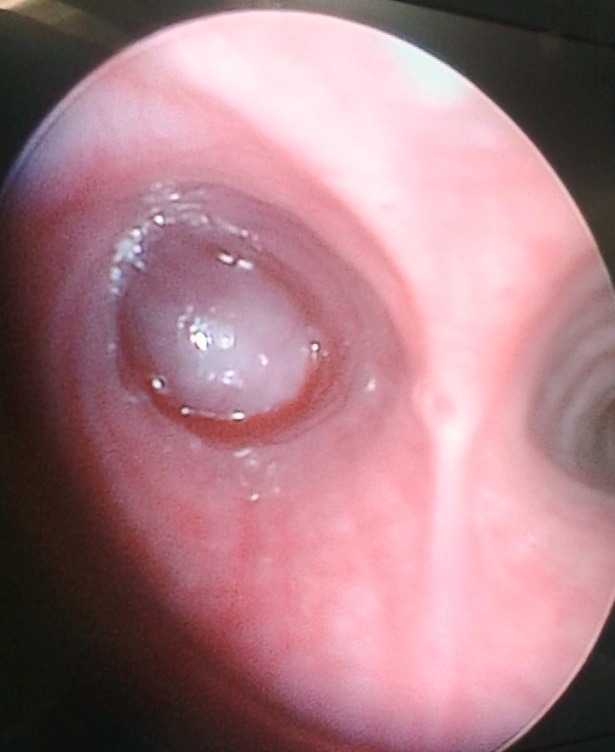
Figure 2:Mass in the left main bronchus.

**Figure:**
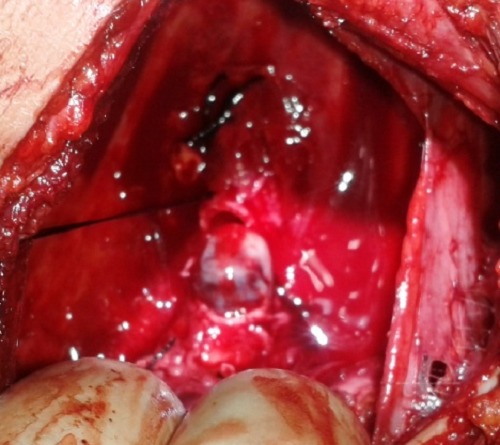
Figure 3:Intraoperative view showing tumor bulging out from left main bronchus.

## DISCUSSION

The most common presentation of bronchial carcinoid in children is hemoptysis, cough, dyspnea, chest pain and pneumonia.[2-4] Our patient had an unusual presentation of choking with hemoptysis which may be related to large amount of bleeding from lesion. These are often diagnosed late. Our case had a delay of five months before diagnosis. The CT scan/MRI can give a clue about mass.[5,6] Bronchoscopic biopsy though useful could be non-representative or insufficient sampling could result in different histopathological diagnosis as happened in the index case.

The accepted treatment modality is complete surgical excision with sparing of all normal tissue. There are reports of endobronchial laser ablation of pedunculated lesions but it may be incomplete due to invasion of bronchial wall by tumor.[1]We planned for lobectomy but owing to extensive disease involving lower lobe as well, pneumonectomy was done.

## Footnotes

**Source of Support:** Nil

**Conflict of Interest:** None declared

